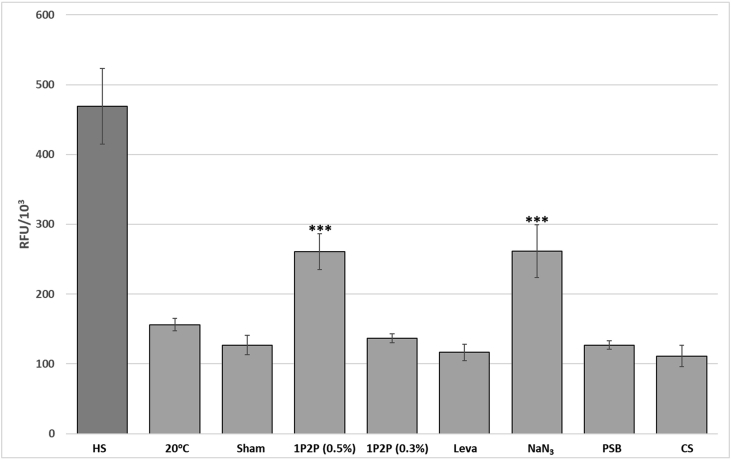# Stress and timing associated with *Caenorhabditis elegans* immobilization methods

**DOI:** 10.1016/j.heliyon.2020.e04263

**Published:** 2020-07-04

**Authors:** Jacob R. Manjarrez, Roger Mailler

**Affiliations:** University of Tulsa, 800 S. Tucker Dr., Tulsa, OK, 74104, USA

**Keywords:** Genetics, Gene expression, Gene regulation, Promoter, Dose-response relationship, Oxidative stress, *Caenorhabditis elegans*, Anesthetic, Stress reporter, Immobilization, Sodium azide, Levamisole

## Abstract

**Background:**

*Caenorhabditis elegans* is a model organism used to study gene, protein, and cell influence on function and behavior. These studies frequently require *C. elegans* to be immobilized for imaging or laser ablation experiments. There are a number of known techniques for immobilizing worms, but to our knowledge, there are no comprehensive studies of the various agents in common use today.

**New method:**

This study determines the relationship between concentration, immobilization time, exposure time, and recovery likelihood for several immobilization agents. The agents used in this study are 1-Phenoxy-2-propanol, levamisole, sodium azide, polystyrene beads, and environmental cold shock. These tests are conducted using a humidified chamber to keep chemical concentrations consistent. Each of these agents is also tested to determine if they exhibit stress-related after effects using the *gcs-1*, *daf-16*, *hsp-4*, *hif-1*, *hsp-16.2,* and *tmem-135* stress reporters.

**Results:**

We present a range of quick mount immobilization and recovery conditions for each agent tested. This study shows that, under controlled conditions, 1-Phenoxy-2-propanol shows significant stress from the *daf-16* reporter. While 1-Phenoxy-2-propanol and sodium azide both create stress related after effects with long term recovery in the case of the *hsp-16.2* reporter.

**Comparison with existing method(s):**

This study shows that commonly used concentrations of immobilizing agents are ineffective when evaporation is prevented.

**Conclusions:**

To improve reproducibility of results it is essential to use consistent concentrations of immobilizing agents. It is also critically important to account for stress-related after effects elicited by immobilization agents when designing any experiment.

## 1 Introduction

Since its introduction in the early 1970s, *Caenorhabditis elegans* has proven to be a tremendously useful model organism [1]. One of its key advantages for use as a model organism is that it is transparent by nature making it an ideal candidate for microscopic examination. Because *C. elegans* remain in a virtually constant state of locomotion, performing multiple series images or laser ablations makes immobilization techniques necessary. For many years, *C. elegans* imaging studies have utilized anesthetics such as sodium azide, levamisole, and 1-Phenoxy-2-propanol to accomplish this task [2, 3, 4, 5].

Although the mode of action for these chemical anesthetizing agents has been studied, there is still a consistent lack of understanding about the relationship between concentration, exposure time, and recovery time. Sodium azide, for example, has been shown to be a potent inhibitor of mitochondrial respiration. It acts by blocking cytochrome c oxidase's interaction in the oxygen reduction site of the electron transport chain [6, 7]. It also interferes with the mitochondrial F-ATPase ATP hydrolase activity, but does so without compromising its synthetic activity [6]. However, this inhibition extends outside the isolation of the mitochondrial F-ATPase to other ATPases with similar protein structures [6]. The anesthetic effect following sodium azide exposure is conceivably linked to the depletion of ATP stores necessary to drive locomotion.

Levamisole works by activating L-type acetylcholine receptors (AChR) channels present in a variety of neurons and muscles [8, 9, 10]. These channels are comprised of five genes: the α subunits *unc-38*, *unc-63*, and *lev-8* and the β subunits *unc-29* and *lev-1* [8,9]. Levamisole is believed to bind at the interface of the α/β subunits causing the channels to open [8, 9] Once open, Ca^2+^ enters the cells leading to spastic paralysis due to continuous stimulation.

There is less information available concerning the mode of action for 1-Phenoxy-2-propanol (1P2P). What little is known comes from the study of its effects on gastropods where 1P2P was shown to reversibly stop neural activity through the elimination of action potentials [11]. Its application has been linked to a reduction in muscle contraction force, which has been correlated to a reduction in muscle cell excitability [11]. However, the direct mode of action for 1P2P remains unknown.

Additionally, little is understood about the stress-related after effects of using these common chemicals even though stress in known to impact various *C. elegans* behaviors and functions [12, 13, 14, 15, 16]. The one exception is that sodium azide is known to produce oxidative stress when used at high concentrations. This effect has been advantageously exploited to study oxidative stress in *C. elegans* [5, 17].

Beyond the use of chemical agents, another method that appears in the literature is temporary immobilization of worms with a short incubation at 4 °C, i.e. cold shock. This technique works by slowing metabolic processes to the point that the worm is no longer able to move. However, exposure time and temperature have a significant impact on the survival of the worm and internal integrity of the structures within it [18, 19]. As a result, exposure time was reduced and temperature increased when compared to the previous studies to improve survival rates [18, 20].

More recently, immobilization techniques that utilize restrictive physical forces rather than chemical agents have also been developed. The two techniques that have become quite widespread are polystyrene microbeads and microfluidics. Both of these techniques allow for extended live imaging [21, 22, 23, 24, 25, 26, 27, 28, 29, 30], but still suffer from a lack of information relating to timing and stress-related after effects.

Polystyrene microbeads work by encapsulating the worm with small objects to increase friction. When the density of these objects gets high enough, the worm is unable to generate enough force to move. Microfluidics work by using pressure to hold the worm in place within a small channel. Microfluidics are being designed for many different applications from single to multi worm, but quick mount and recovery has not been a primary goal [23, 24, 25, 26, 27]. Because of the lack of commercially available sources, the cost associated with implementing a system, the limited use of a single unit, along with the difficulty in individual worm recovery [31], they are not utilized in this study.

To rectify the gap in knowledge about their use, this study provides a detailed investigation into the use of three chemical, one physical, and one temperature technique for immobilizing worms. These immobilization techniques were also evaluated for their induction of stress responsive genes including *hsp-4* [32], *daf-16* [33], *hif-1* [34], *gcs-1* [35], *hsp-16.2* [36] and *tmem-135* [37]. The investigation into these six different stress responsive genes looked into known stress-related processes: 1.) The unfolded protein response (UPR) involved in ER stress [32], 2.) FOXO related stress [33], 3.) Hypoxia-related stress [34], 4.) Phase II detox [35], 5.) Heat and many environmental stresses [36] and 6.) Cold shock [37]. These stress reporters provide an insight into the stress-related after effects resulting from the use of each of these agents.

## 2 Materials and methods

### 2.1 *C. elegans* growth conditions and strains

*C. elegans* were grown at 20 °C on NGM lite plates containing streptomycin and nystatin; seeded with OP50-1 as a bacteria food source [1]. All experiments were completed with young adults, age-synchronized by picking L4 stage animals to fresh food Plates 12–24 h before the experiment. Strains used in this study were N2, UL1447, TJ356, SJ4005, LD1171, CL2070, which were sourced from the CGC, and MAB124 [37].

### 2.2 Chemical immobilization

Immobilization consisted of three steps: immobilization, exposure, and recovery. During the immobilization step, worms were placed into a 20μL drop of M9 buffer containing the indicated concentration of anesthetic on a glass microscope slide at room temperature. M9 buffer was selected as a suitable vehicle for all chemical anesthetics, while maintaining hydration during a quick mount and recovery procedure. The slide was placed in a room temperature chamber, with a wet Kimwipe to maintain humidity. Samples were checked every minute for 5 min, then every 5 min until immobilized. Immobilization was scored by the absence of spontaneous movement over a thirty-second period, this is the determined immobilization time.

Once determined to be immobilized, exposure time, the total time the worms are exposed to the agent once immobilized, was simulated by incubating the worms in the humidified chamber for the duration of this step. Due to its prolonged recovery time, worms treated with levamisole skipped the exposure step and were directly moved to recovery.

At the conclusion of the exposure time, 100 μL of M9 buffer was added to the 20 μL drop to allow for retrieval from the glass slide. The worms were transferred to a 100 μL drop of M9 buffer on a seeded NGM lite plate. The plates were incubated at 20 °C and checked every 15 min until the worms completely recovered. Recovery was scored by the presence of spontaneous movement or full body movement when lightly prodded with a platinum worm pick.

The anesthetics and concentrations used in this study are as follows: 1.) 1-Phenoxy-2-propanol (≥95.0%TCI): 0.2% (13.97mM); 0.3% (20.92mM); 0.4% (27.87mM); 0.5% (34.82mM); 0.6% (41.84mM); 0.7% (48.79mM); 0.8% (55.74mM); 0.9% (62.69mM); & 1.0% (69.71mM) 2.) Levamisole (in mM) (TCI): 0.25; 0.5; 1.0; 3.0 & 5.0. 3.) Sodium Azide (in mM) (Sigma): 10; 20; 50; & 100.

### 2.3 Polystyrene microbeads immobilization

**Figure 1:**
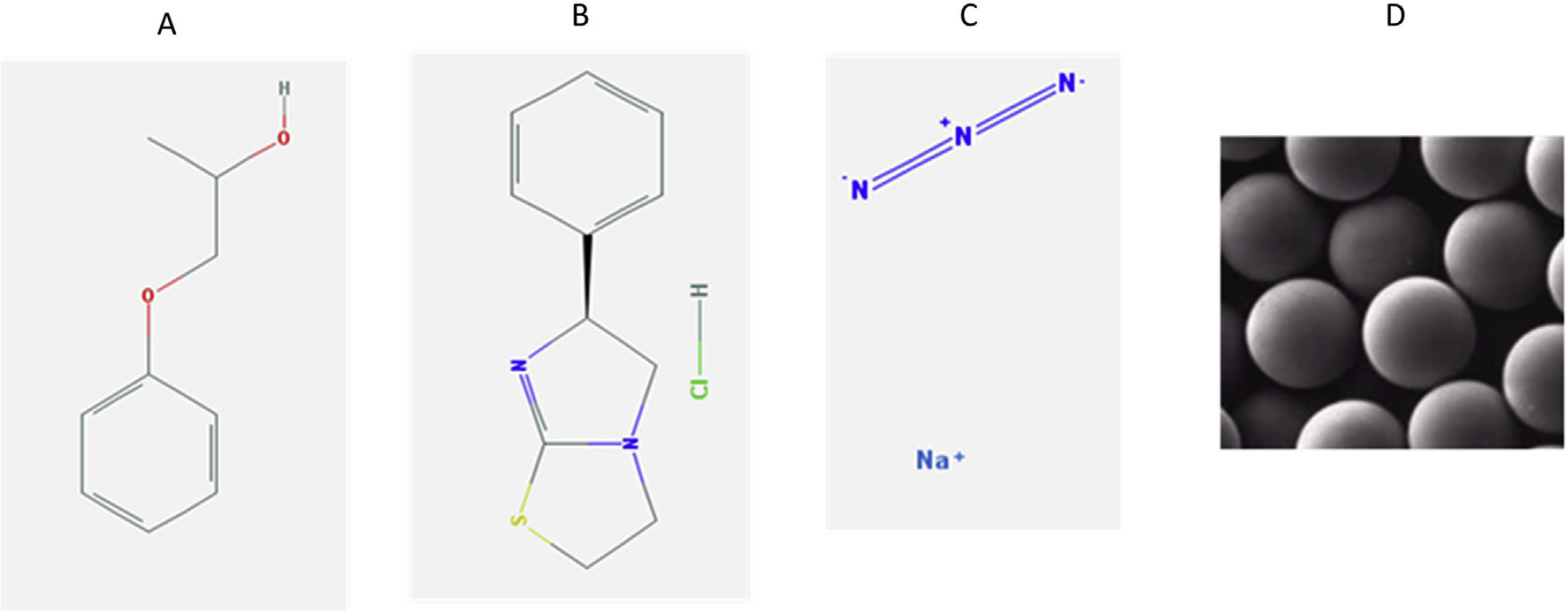
Chemical and Physical Structures of immobilization agents. A. 1-Phenoxy-2-propanol [61] B. Levamisole HCl [62]C. Sodium Azide [63] D. 0.10μM Polystyrene microspheres [38].

For the immobilization step, agarose pads were created by applying 45–50 μL of 5% agarose to a slide prior to compression [3, 21]. The 5% agarose pads were used because 3% pads allowed cavities to development during the exposure time while 7% pads increased the amount of ruptures prior to recovery. The 0.10 μm polystyrene microbeads (PSB) were then placed onto the center of the agarose pad. Worms were individually rinsed in a 70 μL spot of M9 buffer on a plain glass slide for 1 min to remove any *E. coli* and then added to the suspension of microbeads. To determine the volume needed for immobilization, we tested 3μL [21], 5 μL [21], 7 μL, and 10 μL of the commercial available suspension [38] (Figure 1).

Once all the worms, 5 worms per pad, were added to the PSB, the exposure time was started by gently placing a coverslip (22 × 22 mm, 0.13–0.17 mm thickness) onto the agarose pad. At the end of the exposure period, the worms were recovered using the worm recovery procedure outlined below.

### 2.4 Cold shock immobilization

Cold shock was carried out on 5% agarose pads, made as previously described. Prewashed worms, 5 worms per pad, were transferred to 9 μL M9 in the center of the agarose pad. After adding the worms, the exposure step began by adding a coverslip and incubating the slide at 4 °C for 30, 45, 60, 90, or 120 min. The slide was then moved to room temperature and maintained continuously on a standard microscope stage. The worms were checked every minute for 5 min, then every 5 min for spontaneous movement. At the end of the exposure period the worms were recovered using the worm recovery procedure outlined below.

### 2.5 Recovery from agarose pads

Before the mounting liquid dried or microbeads dehydrated, the coverslip was carefully removed with #7 forceps in an upward motion [3, 21]. M9 was then added to the pad and the worms were picked onto a 100 μL spot of M9 on a seeded plate [22].

### 2.6 Microscopy and imaging

Images were acquired using a Leica DMi8, X-Cite XLED1 (Lumen Technologies), Zyla-4.2P-CL10 sCMOS camera (Andor), and in-house software. Images were captured using a 10x objective and an exposure time of 9.8 ms. Relative fluorescence intensity (RFU) was calculated using ImageJ (NIH) by subtracting the average background fluorescence from the fluorescence intensity of the worm's body. Images were processed using Adobe Photoshop and Illustrator with statistical analysis completed using Microsoft Excel.

### 2.7 Stress assays

Immobilization was carried out using the best concentration, volume, or time for the technique being evaluated. Unless otherwise noted, the samples were transferred to a 5% agarose pad with the indicated solution for the duration of the exposure time and then recovered prior to imaging. All stress related imaging experiments were carried out using PSB stabilization to limit variation in immobilization times and image quality. When exposed to levamisole, worms were immobilized according to Table 3 and then immediately placed on an agarose pad for 30 min in M9 prior to recovery or imaging. The strains were referenced against the positive controls indicated for each strain. Unless otherwise noted, negative control for all stress related assays were sham, M9 mounted samples, along with uninterrupted 20 °C grown worms.

#### 2.7.1 ER stress

The upregulation of the HSP-4::GFP reporter in the intestine of the positive control worms were induced by heat shock and used as an indicator of ER stress [39]. A one hour heat shock at 35 °C with a 24 h recovery at 20 °C was used as a positive control, modified from Bischof et al., 2008 [39].

#### 2.7.2 DAF-16 (FOXO) stress response

The translocation of the DAF-16::GFP reporter from cytoplasmic to nuclear localization in the hypodermal cells was used as an indicator of stress activation. A one hour heat shock at 37 °C with a two hour recovery at 20 °C was used as a positive control, modified from Gerke et al., 2014 [33].

#### 2.7.3 Hypoxia-inducible factor

The upregulation of the HIF-1::GFP reporter in positive control worms was induced by hypoxia according to Hong et al., 2004 [34]. Each immobilization technique was performed and samples were imaged immediately to prevent the rapid degradation of HIF-1 induction upon reoxygenation [40].

#### 2.7.4 Heat-shock factor

The induction of hsp-16.2p::GFP was used to indicate heat shock and other environmental stresses. A three hour heat shock at 35 °C with a one hour recovery at 20 °C was used as a positive control, modified from Strayer et al., 2003 [36]. An extended recovery time was also used according to Rea et al., 2005, which used a recovery time of 16 h at 20 °C after exposure [41].

#### 2.7.5 Cold shock

Fluorescence induction of TMEM-135::GFP by incubation at 4 °C for two hours was used as a positive control for this assay, modified from Exil et al., 2010 [37].

#### 2.7.6 Phase II detox stress

The induction of the gcs-1p::GFP in positive control worms was used to indicate Phase II detox *skn-1* activation. The *gcs-1* reporter was chosen because, unlike *gst-4*, it can only be activated by *skn-1* [42]. A one hour heat shock at 37 °C with a four hour recovery at 20 °C was used as a positive control, modified from An et al., 2003 [35].

## 3 Results

### 3.1 Immobilization by 1-Phenoxy-2-propanol

**Figure 2:**
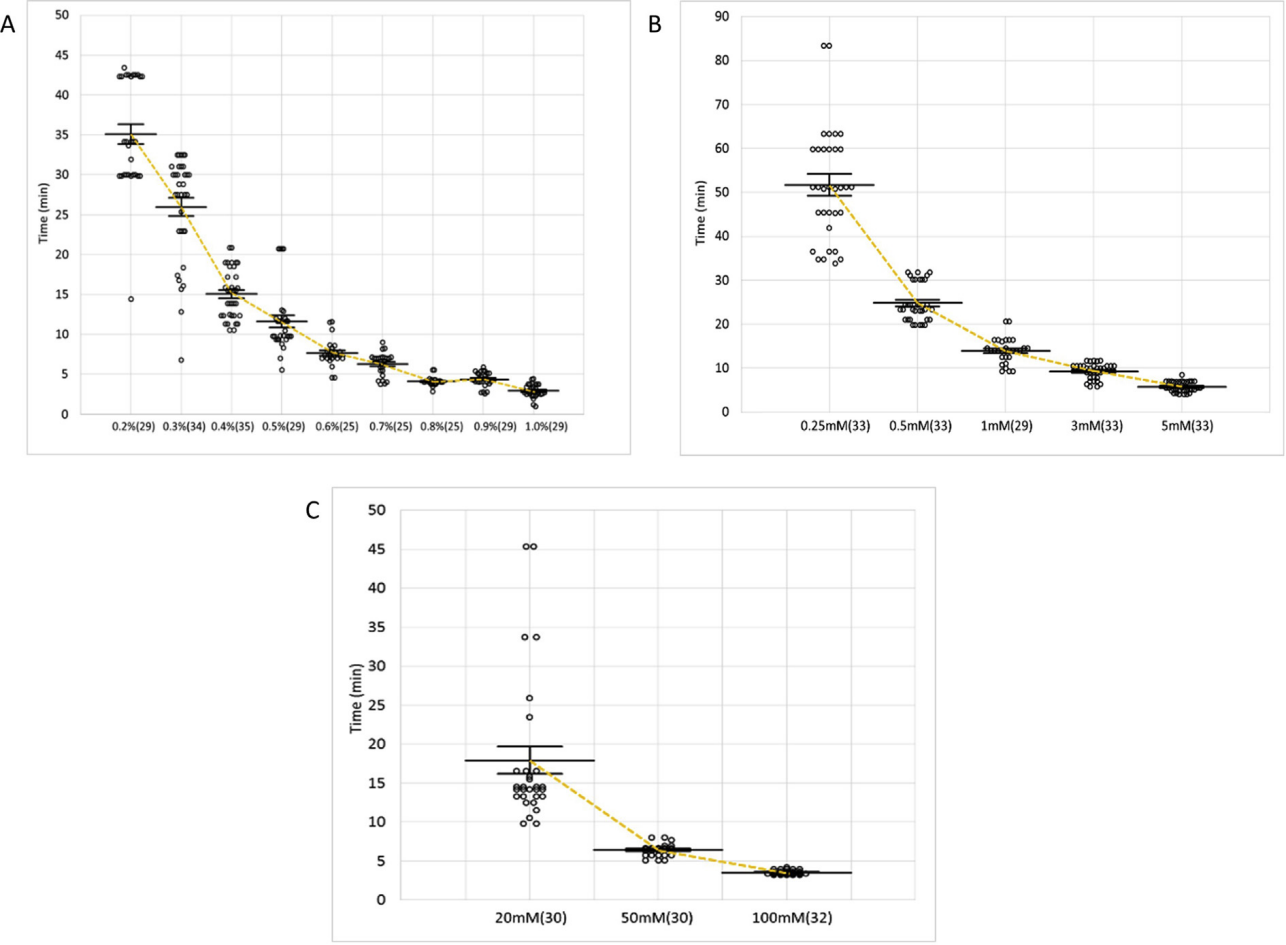
The immobilization time of wild-type (N2) worms. A. 1P2P B. Levamisole and C. Sodium Azide. Solid bars show the mean and standard error for each concentration. The number of worms tested at each concentration is indicated in parentheses.

Immobilization with 1P2P progressed in a concentration-dependent manner from 0.2% - 1.0% as seen in Figure 2A. The time to complete immobilization was from 35 min for 0.2% to just under 3 min for the highest concentration 1.0%. Because only 5% of the animals were immobilized after 3 h of exposure, 0.1% 1P2P was not considered further in this study.

The worms immobilized into a smooth and elongated resting state, but did not show signs of overt stress such as rupturing through the vulva at any concentration. However, at higher concentrations or after lengthy exposure (S1 Table), a significant number worms did not recover. Therefore, the exposure times were adjusted to maximize imaging time while still retaining the ability to recover the worms for further experimentation (Table 1).

**Table 1:** Summary of data for 1P2P.

1-Phenoxy-2-propanol				
Concentration	Immobilization	Exposure	Recovery	Recovery Rate
0.2% (13.97mM)	35.1 ± 1.27 min (29)	30min	64.5 ± 4.35 min (20)	74% (27)
0.3% (20.92mM)	26.0 ± 1.13 min (34)	30min	69.0 ± 3.91 min (31)	100% (31)
0.4% (27.87mM)	15.1 ± 0.53 min (35)	20min	63.6 ± 4.69 min (23)	82% (28)
**0.5% (34.82mM)**	**11.6 ± 0.75 min (29)**	**15min**	**71.4 ± 5.46 min (27)**	**100% (27)**
0.6% (41.84mM)	7.7 ± 0.33 min (25)	15min	107.1 ± 6.90 min (19)	86% (22)
0.7% (48.79mM)	6.3 ± 0.30 min (25)	10min	112.6 ± 8.00 min (19)	86% (22)
0.8% (55.74mM)	4.1 ± 0.11 min (25)	10min	114.8 ± 9.34 min (18)	82% (22)
0.9% (62.69mM)	4.4 ± 0.16 min (29)	5min	88.7 ± 4.44 min (24)	92% (26)
1.0% (69.71mM)	2.9 ± 0.15 min (29)	5min	85.4 ± 5.04 min (21)	85% (26)

Values are given as mean ± SEM along with a recovery rate for each concentration tested. The number of worms tested at each concentration is indicated in parentheses. **Bold** indicates best concentration.

**Figure 3:**
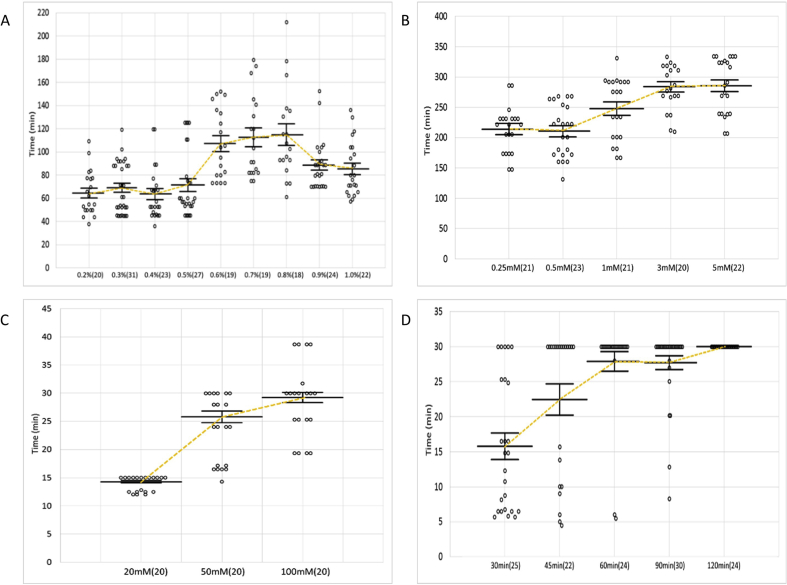
The recovery time of wild-type (N2) worms from the immobilization. A. 1P2P *B. levamisole* C. Sodium Azide and D. Cold Shock. The immobilization from 4 °C incubation was terminated at 30 min to ensure recovery of the worms. Solid bars show the mean and standard error for each concentration. The number of worms tested at each concentration is indicated in parentheses.

Recovery takes between one to two hours depending on the concentration. The overview of recovery across the concentration range is outlined in Figure 3A. As the figure shows, even with reduced exposure, higher 1P2P concentrations require an extended amount of recovery time. For example, at the highest concentrations of 0.9% and 1.0%, it took worms over one hour and twenty minutes to fully recover (Table 1).

The best concentration for 1-Phenoxy-2-propanol (1P2P) is 0.5% and is used for the stress-related immobilization experiments. This concentration was chosen because it has a 100% recovery rate, a reasonable immobilization and exposure time, and a modest recovery period (Table 1). However, if a 100% recovery rate is not required then a quick immobilization with a high percentage 1P2P followed by immediate mounting can provide adequate imaging time during the recovery. This is similar to the levamisole protocol outlined in the methods section.

### 3.2 Immobilization by levamisole

The levamisole immobilization time decreases in an inversely proportional manner with increases in concentration (Figure 2B). Unlike 1P2P, worms immobilized with levamisole do not have a smooth and elongated stature, but are hypercontracted near the head with a more relaxed posture further posterior.

Based on tritiated meta-aminolevamisole experiments, it has been shown that levamisole has a extended half-life in *C. elegans* [10]. Our expeirments found that prolonged exposure to this agent causes exceedingly long recovery times or death. Therefore, we eliminated the exposure step in our experiments. However, immediate removal did not revive the worm and recovery was still a prolonged process. In fact, recovery times only slightly decrease when reducing the concentration, Figure 3B.

For the purposes of the stress-related experiments, we used the 1mM levamisole concentration because it has a 100% recovery and reasonable immobilization time of 14 min (Table 2). It should be noted that exposure to levamisole results in the release of a large number of eggs. If subsequent experimentation involves egg laying or brood size, this immobilization method should be avoided.

**Table 2:** Summary of data for Levamisole.

Levamisole			
Concentration	Immobilization	Recovery	Recovery Rate
0.25mM	51.68 ± 2.51 min (33)	213.96 ± 9.39 min (21)	100% (21)
0.5mM	24.83 ± 0.75 min (33)	210.48 ± 8.96 min (23)	92% (25)
**1mM**	**13.99 ± 0.54 min (29)**	**247.94 ± 11.17 min(21)**	**100% (21)**
3mM	9.29 ± 0.32 min (33)	283.81 ± 8.45 min (20)	80% (25)
5mM	5.73 ± 0.21 min (33)	285.25 ± 9.66 min (22)	88% (25)

Values are given as mean ± SEM along with a recovery rate for each concentration tested. The number of worms tested at each concentration is indicated in parentheses. **Bold** indicates determined best concentration.

### 3.3 Immobilization by sodium azide

Like both 1P2P and levamisole, immobilization time for sodium azide was inversely proportional to concentration, Figure 2C. The time to complete immobilization ranged from about 4 min at 100mM to just under 20 min at 20mM (Table 3). At 10mM, which is frequently referenced in the literature, just 42% of the worms were immobilized within 3 h in a humidified environment [3, 43, 44, 45, 46, 47, 48, 49, 50, 51]. This suggests 10mM is ineffective at immobilizing worms unless it is allowed to become concentrated through evaporation. We did not consider this concentration further.

**Table 3:** Summary of data for sodium azide.

Sodium Azide				
Concentration	Immobilization	Exposure Time	Recovery	Recovery Rate
**20mM**	**17.94 ± 1.73 min (30)**	**30**	**14.29 ± 0.22 min (20)**	**100% (20)**
50mM	6.46 ± 0.13 min (30)	30	25.82 ± 1.04 min (20)	100% (20)
100mM	3.56 ± 0.05 min (32)	30	29.22 ± 0.91 min (20)	100% (20)

Values are given as mean ± SEM along with a recovery rate for each concentration tested. The number of worms tested at each concentration is indicated in parentheses. **Bold** indicates determined best concentration.

Once immobilized, the worms had a smooth and elongated resting state, similar to 1P2P, and showed no signs of overt stress. A common exposure time of 30 min yielded a significant number of recoverable worms for all sodium azide concentrations.

Recovery time was not concentration dependent and occurred rapidly. The worms immobilized with 20mM recovered within 15 min where 100mM recovered in under 30 min, Figure 3C. This illustrates a more precise recovery period than was previously reported [52]. A full summary of the immobilization, exposure, recovery times, and recovery rates for sodium azide are shown in Table 3.

The 20mM concentration was considered the best for the stress-related experiments because it has a 100% recovery rate, reasonable immobilization and exposure time, and a rapid recovery (Table 3). Additionally, sodium azide is known to cause stress-related effects such as *skn-1* induced oxidative stress, chemical induce hypoxia, and induction of stress proteins at higher concentrations or extended exposure times [5, 35, 53, 54]. Therefore, keeping the concentration low and maintaining a shorter exposure time should reduce or eliminate these unwanted after effects [5].

### 3.4 Immobilization by polystyrene beads

Unlike chemical immobilization, PSB works by physically restraining the worm. However, we found that PSB do not completely immobilize the worm as the head and tail still retain limited freedom. Therefore, we refer to this technique as a stabilization method.

The primary consideration when using this method is drying. Although not lethal for the worms, drying makes it considerably more difficult to recover the worms after the exposure step. In our experiments, we found the size of the agarose pad, in combination with the volume of PSB, determine the drying rate. For example, we found that 3 and 5μL volumes covered an insufficient area of our agarose pads, which did not create enough surface tension with the coverslip to prevent evaporation. A volume of 7 μL covered a larger area and maintained a better seal allowing the pad to remain moist, although not wet. A volume of 10 μL created a great deal of fluidity and made it difficult to keep the worms on the agarose pad when placing the cover glass.

The 7 μL volume was considered the best for the stress-related experiments because it has a 100% recovery rate and recovery was fairly easy after 30 min of exposure (Table 4). If a longer stabilization is required this can be obtained in a number of ways: 1.) using an alternative hydration vehicle other than M9, 2.) sealing the coverslip while using PSB [22] 3.) using hydrogel [55, 56, 57, 58] or 4.) using whichever microfluidics platform is reasonable and can be obtained for your study [23, 24, 25, 26, 27, 28, 29, 30].

**Table 4:** Summary of data for polystyrene beads.

Polystyrene beads		
Volume	Immobilization Time	Recovery Rate
3μL	30 min (25)	100%
5μL	30 min (25)	100%
**7μL**	**30 min (25)**	**100%**
10μL	30 min (25)	100%

Immobilization on 5% agarose pads with an indicated recovery rate for each volume tested. The number of worms tested at each concentration is indicated in parentheses. **Bold** indicates determined best volume (optimized for a 45–50 μL agarose pad.).

### 3.5 Immobilization by cold shock

The primary concerns for using cold shock as an immobilization technique are rupturing caused by rapid changes in temperature and imaging time once the worm is moved to room temperature. Table 5 and Figure 3D summarize the results. The cold shock incubation time with the best recovery rate combined with the longest imaging time was 90 min at 4 °C, Table 5. Incubation for 60 min had a similar immobilization time, but suffered from an increase in worm ruptures. Therefore, the 90-minute incubation was selected as the best condition for the stress-related experiments. Like PSB, the head and tail regions retain a limited degree of movement in some cases, but are significantly slowed compared to room temperature M9 mounts. As time proceeds, the worms progress toward full movement as they equilibrate to room temperature.

**Table 5:** Summary of data for cold shock.

Cold Shock		
Incubation Time at 4 °C	Time remaining Immobilized After Room Temperature Shift	Recovery Rate
30 min	15.78 ± 1.89 min (25)	100%
45 min	22.45 ± 2.23 min (33)	67%
60 min	27.90 ± 1.40 min (27)	89%
**90 min**	**27.71 ± 0.98 min (30)**	**100%**
120 min	30 ± 0 min (28)	86%

Values are given as mean ± SEM along with a recovery rate after each cold shock incubation time tested. The number of worms tested at each concentration is indicated in parentheses. **Bold** indicates determined optimal incubation time.

### 3.6 Immobilization of males

The immobilization of N2 males took nearly half the time needed for hermaphrodites for all chemical agents (S2 Table). Recovery times were reduced by half for 1P2P and levamisole, but slightly increased when using sodium azide (S2 Table). Cold shock was an ineffective means of immobilizing male worms as they recovered rapidly once shifted to room temperature. The males regained complete movement 3 times faster than hermaphrodites (S2 Table.)

PSB was not successful either. The smaller size and more active male body makes this method unreliable. Males also seem to take particular advantage of abnormalities in the agarose pad surface to escape capture. However, when captured the male tail seems to be very well controlled by this technique, even when the head retains some freedom of movement.

### 3.7 Stress reporters

**Figure 4:**
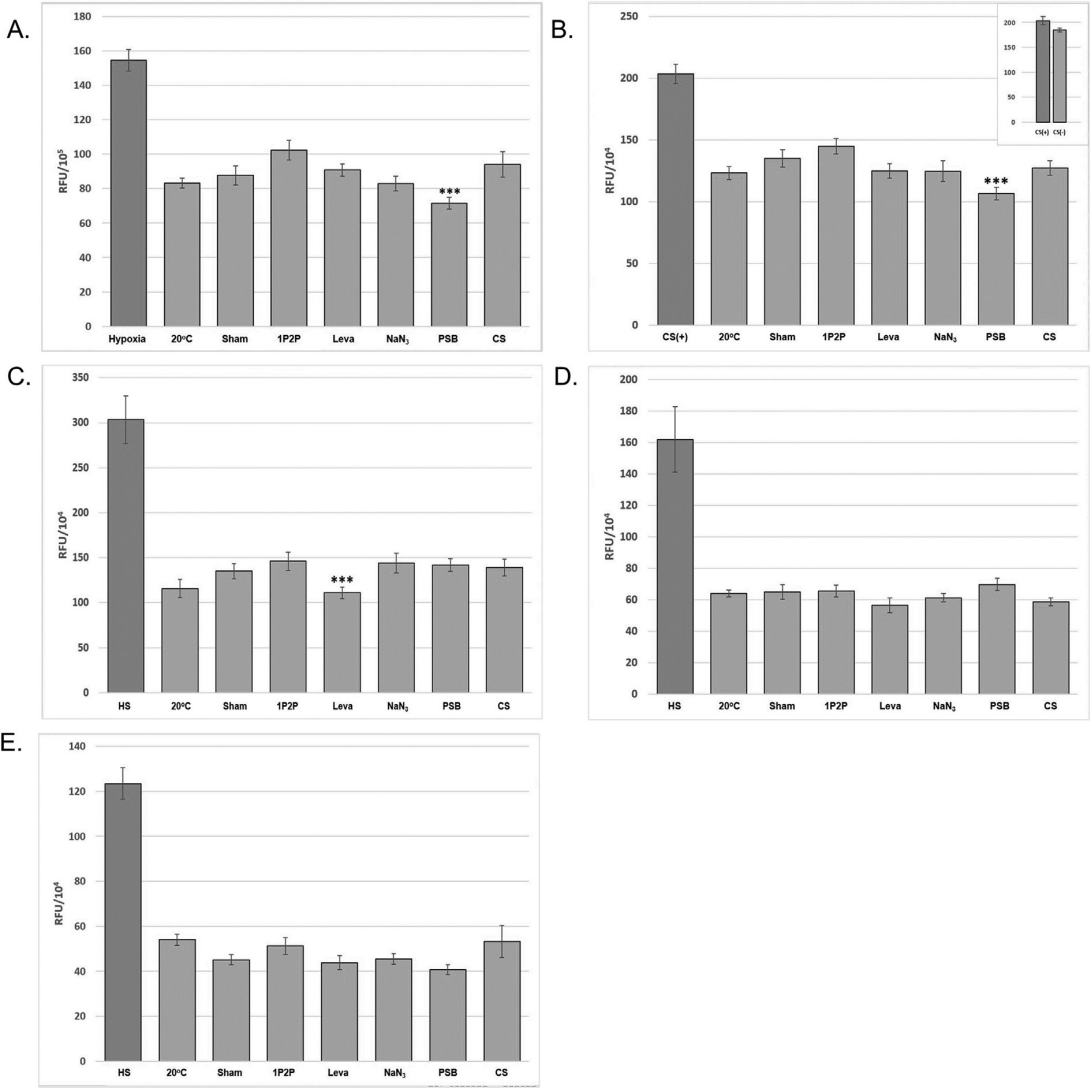
The relative level of integrated fluorescence induced under optimal immobilization conditions. A. HIF-1::GFP. Hypoxia was used as a positive control. Each sample was imaged immediately after exposure due to the transient nature of *hif-1* signaling [40]. B. TMEM-135::GFP. Cold shock was used as the positive control. Each sample was imaged immediately after exposure. The inset shows CS (+) [2hr cold shock] vs CS (w/o) without room temperature post immobilization exposure. C. HSP-4::GFP. Each was allowed to recover for 24 h at 20 °C prior to imaging. D. hsp-16.2p::GFP. Heat shock was used as a positive control. Each was allowed to recover for 1 h at 20 °C prior to imaging. E. gcs-1p::GFP. Heat shock was used as a positive control. Each sample was allowed to recover for 4 h at 20 °C prior to imaging. (∗∗∗) denotes a two-tailed Student's *t*-test p-value < 0.05 and n = 25 was used in all assays.

The HIF-1::GFP stress reporter was used to indicate hypoxic stress. There was no *significant increase* under any of the immobilization conditions tested, Figure 4A. However, 0.5% 1P2P shows a HIF-1::GFP response that is slightly below the 95% CI (P = 0.071). So, care should be taken when using 0.5% 1P2P because *hif-1* induced stress could influence subsequent experiments. It is interesting to note that using PSB actually significantly reduced hypoxic stress when compared to the sham control.

The TMEM-135::GFP reporter was utilized as an indicator of cold stress and was not shown to be increased under the tested immobilization conditions, Figure 4B. Like *hif-1*, TMEM-135::GFP reporter induction degrades rapidly so, as the inset shows, if the sample is imaged immediately upon removal from 4 °C., a clear induction is present. However, no other condition appears to elicit a response when adhering to the stated protocols. Interestingly, using PSB reduced the response to lower levels than that of the sham control, Figure 4B.

The HSP*-4*::GFP stress reporter was utilized as an indicator of the UPR. However, it was not shown to increase under the tested immobilization conditions and, in the case of levamisole, was lower than the sham control, Figure 4C.

The hsp-16.2p::GFP stress reporter was utilized as an indicator of environmental stress and was not shown to increase under any of the tested immobilization conditions, Figure 4D. When allowed to recover for an extended period of time, there was an increase in hsp-16p fluorescence for both the 20 mM sodium azide and 1P2P at 0.5%, Figure S1 [36,41]. Given that the lower concentration of sodium azide does not successfully immobilization *C. elegans*, this conditions should be avoided if environmental stress is a concern for your downstream application. Because 0.3% 1P2P also had a 100% recovery rate, it was tested as a substitution. It was shown to have a nominal response compared to the sham control, Figure S1. Therefore, this concentration of 1P2P can be a viable alternative for your experiment to alleviate *hsp-16.2* stress related after effects.

The gcs-1p::GFP stress reporter was utilized as an indicator of skn-1 activation of the Phase II detox system. The *gcs-1* reporter was not shown to be increased under any of the tested immobilization conditions, Figure 4E.

**Figure 5:**
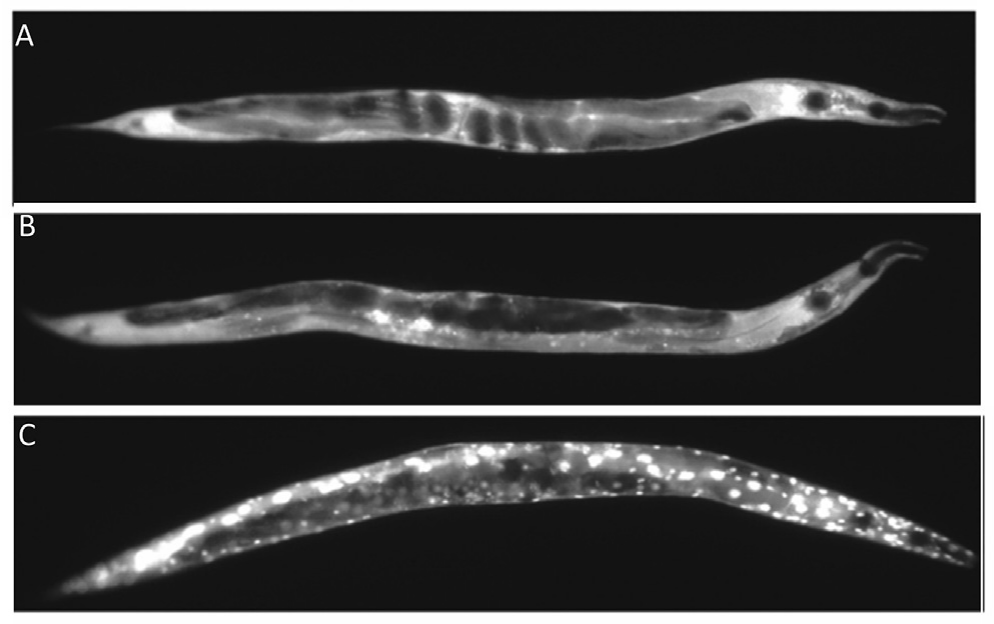
DAF-16::GFP induction categories. From top to bottom is the hypodermal translocation patterning associated with the A. low, *B. medium* and C. high categories referenced in Table 6.

The DAF-16::GFP reporter shows FOXO related stress. Samples were scored into one of three categories based on the amount of cytoplasmic translocation of DAF-16::GFP. “Low” refers to animals where DAF-16::GFP was predominately dispersed in the cytoplasm of the hypodermal cells, as shown in Figure 5A. “Medium” refers to animals where nuclear translocation of DAF-16::GFP was present at anterior, midbody, or posterior, but was not distributed throughout the body, as in Figure 5B. “High” indicates a very strong DAF-16::GFP translocation into hypodermal nuclei throughout the body, as in Figure 5C.

For most of the tested conditions, there were no significant indication of FOXO related stress. The two exceptions were a minor increase (<3%) in the number of samples scored in the medium category when using PSB, and a significant increase (35%) in the number scored as high when using 0.5% 1P2P, Table 6.

**Table 6:** Summary of DAF-16::GFP translocation under various immobilization conditions.

DAF-16::GFP				
Condition	Low	Medium	High	*n*
Heat	0.00%	1.75%	98.25%	57
20 °C	80.70%	17.54%	1.75%	53
Sham	81.13%	13.21%	5.66%	57
1P2P 0.5%	40.00%	20.00%	40.00%	25
1P2P 0.3%	78.57%	17.85%	3.57%	28
Levamisole	93.94%	6.06%	0.00%	33
NaN_3_	86.67%	10.00%	3.33%	30
PSB	84.00%	16.00%	0.00%	25
Cold	92.86%	7.14%	0.00%	28

One day old adult TJ356 transgenic worms were exposed to the indicated conditions. Sham refers to the M9 control incubation for the immobilization experiments. Values are given as mean ± SEM. *n* indicates the number of animals analyzed for each condition.

One possible way to reduce FOXO related stress while using 1P2P is to reduce the concentration. In an additional experiment, 0.3% 1P2P was tested for the DAF-16::GFP response. When used according to the schedule in Table 1, there was a 36.43% decrease in the number of *daf-16* positive samples scored as high putting 0.3% 1P2P below the sham control level in this category.

## 4 Discussion

Immobilization of *C. elegans* has been performed for many years, but researchers have lacked a definitive guide to help them select a proper technique. The techniques tested in this article cover many of the methods that are commonly used, cost effective, and allow easy recovery of specimens [1, 2, 3, 4, 5, 21, 22].

The inclusion of concentration and time parameters used for immobilization during experimentation is an important step towards resolving reproducibility issues found in *C. elegans* and scientific literature in general [59, 60]. For example, this work discovered that two of the most commonly used anesthetics, 0.1% 1P2P and 10% sodium azide, were ineffective when evaporation was slowed using a humidified chamber. This suggests that previous research used these agents at higher concentrations than reported. It also suggests that it is important to use a humidified chamber during the immobilization step to maximize imaging time, minimize the deleterious effects of the chemical, and produce consistent results.

The scientific literature related to the stress response associated with immobilization agents is also limited. This study just begins to scratch the surface for a series of well-known and widely used immobilization agents and stress response genes. This work discovered, for instance, that elevated concentrations of 1P2P (>0.5%) caused both an *hsp-16.2* and *daf-16* stress-related response. Knowing this, the protocol was readjusted to minimizing the stress-related impact by lowering the concentration and increasing the immobilization time. This demonstrates that stress-related after effects elicited by immobilization agents must be accounted for in the design of any experiment, whether it is solely imaging or includes a downstream application.

## Declarations

### Author contribution statement

Jacob R. Manjarrez: Conceived and designed the experiments; Performed the experiments; Analyzed and interpreted the data; Contributed reagents, materials, analysis tools or data; Wrote the paper.

Roger Mailler: Contributed reagents, materials, analysis tools or data; Wrote the paper.

### Funding statement

This material is based upon work supported by the 10.13039/100000181Air Force Office of Scientific Research, under award numbers FA9550-15-1-0060 and FA9550-18-1-0308.

### Competing interest statement

The authors declare no conflict of interest.

### Additional information

No additional information is available for this paper.

## Figures and Tables

**FigureS1.jpg:**