# Supplementary material for: Stress and timing associated with Caenorhabditis elegans immobilization methods
Source: Heliyon. 2020 Jul 4;6(7):e04263. doi: 10.1016/j.heliyon.2020.e04263 (PMC7339059; doi:10.1016/j.heliyon.2020.e04263)
Supplement: Supplementary file 1 — SupplementalDocs.docx [file mmc1.pdf]

Supplemental Information

S1 Table. Generalized exposure attempted for 30 minutes for all 1P2P concentrations

1-Phenoxy-2-propanol		
Concentration	Recovery	Recovery Rate
0.2%	91.05 ± 9.63 min (5)	100%
0.3%	121.75 ± 2.75 min (5)	80%
0.4%	94.42 ± 12.05 min (5)	100%
0.5%	159.63 ± 15.33 min (5)	100%
0.6%	192.83 ± 0 min (5)	40%
0.7%	died (5)	0%
0.8%	died (5)	0%
0.9%	died (5)	0%
1.0%	died (5)	0%

Values are given as mean ± SEM along with a recovery rate for each concentration tested. The number of worms tested at each concentration is indicated in parentheses.

S2 Table. Summary of data for wild-type N2 males

N2 Males			
Agent	Immobilization	Recovery	Recovery Rate
1P2P (0.5%)	5.93 ± 0.25 min (25)	31.96 ± 1.65 min (25)	100%
Levamisole (1mM)	11.20 ± 0.63 min (25)	224.96 ± 3.86 min (25)	100%
Sodium azide (20mM)	5.64 ± 0.08 min (25)	19.08 ± 0.17 min (25)	100%
Cold Shock (90min)	10.16 ± 1.06 min (25)	immediate (25)	100%
PSB (30min)	30 min (stabilized) (25)	immediate (25)	100%

Values are given as mean ± SEM along with a recovery rate for each optimal condition tested. The number of worms tested at each concentration is indicated in parentheses
